# Incidence of orthostatic hypotension and cardiovascular response to postoperative early mobilization in patients undergoing cardiothoracic and abdominal surgery

**DOI:** 10.1186/s12893-017-0314-y

**Published:** 2017-11-28

**Authors:** Masatoshi Hanada, Yuichi Tawara, Takuro Miyazaki, Shuntaro Sato, Yosuke Morimoto, Masato Oikawa, Hiroshi Niwa, Kiyoyuki Eishi, Takeshi Nagayasu, Susumu Eguchi, Ryo Kozu

**Affiliations:** 1 Cardiorespiratory Division, Department of Rehabilitation Medicine, Nagasaki University Hospital, 1-7-1 Sakamoto, Nagasaki, 852-8501 Japan; 2 Department of Cardiopulmonary Rehabilitation Science, Unit of Rehabilitation Sciences, Graduate School of Biomedical Sciences, Nagasaki University, 1-7-1 Sakamoto, Nagasaki, 852-8501 Japan; 3 Department of Surgical Oncology, Nagasaki University Graduate School of Biomedical Sciences, 1-7-1 Sakamoto, Nagasaki, 852-8501 Japan; 4 Clinical Research Center, Nagasaki University Hospital, 1-7-1 Sakamoto, Nagasaki, 852-8501 Japan; 5 Division of Thoracic Surgery, Respiratory Disease Center, Seirei Mikatahara General Hospital, 3458 Mikatahara, Hamamatsu, 433-8558 Japan; 6 Department of Cardiovascular surgery, Nagasaki University Graduate School of Medicine, 1-7-1 Sakamoto, Nagasaki, 852-8501 Japan; 7 Department of Surgery, Nagasaki University Graduate School of Biomedical Science, Nagasaki, Japan, 1-7-1 Sakamoto, Nagasaki, 852-8501 Japan

**Keywords:** Orthostatic hypotension, Cardiovascular responses, Early mobilization, Cardiothoracic and abdominal surgery

## Abstract

**Background:**

In cardiothoracic and abdominal surgery, postoperative complications remain major clinical problems. Early mobilization has been widely practiced and is an important component in preventing complications, including orthostatic hypotension (OH) during postoperative management. We investigated cardiovascular response during early mobilization and the incidence of OH after cardiothoracic and abdominal surgery.

**Methods:**

In this prospective observational study, we consecutively analyzed data from 495 patients who underwent elective cardiothoracic and abdominal surgery. We examined the incidence of OH, and the independent risk factors associated with OH during early mobilization after major surgery. Multivariate logistic regression was performed using various characteristics of patients to identify OH-related independent factors.

**Results:**

OH was observed in 191 (39%) of 495 patients. The incidence of OH in cardiac, thoracic, and abdominal groups was 39 (33%) of 119, 95 (46%) of 208, and 57 (34%) of 168 patients, respectively. Male sex (OR 1.538; *p* = 0.03) and epidural anesthesia (OR 2.906; *p* < 0.001) were independently associated with OH on multivariate analysis.

**Conclusions:**

These results demonstrate that approximately 40% patients experience OH during early mobilization after cardiothoracic and abdominal surgery. Sex was identified as an independent factor for OH during early mobilization after all three types of surgeries, while epidural anesthesia was only identified after thoracic surgery. Therefore, the frequent occurrence of OH during postoperative early mobilization should be recognized.

**Trial registration:**

University hospital Medical Information Network Center (UMIN-CTR) number UMIN000018632. (Registered on 1st October, 2008).

## Background

Enhanced recovery after surgery (ERAS) programs, also known as fast-track surgery, are multimodal perioperative programs that aim to accelerate recovery, shorten hospital stay, and reduce postoperative complications. Postoperative management includes epidural anesthesia, early mobilization, early enteral nutrition, and early removal of catheters [1–3]. Early mobilization has been widely practiced and is an important component in preventing complications, including orthostatic hypotension (OH) during postoperative care [4, 5]. However, postoperative patients are frequently exposed to prolonged immobilization. Immobility has an important role in the development of neuromuscular weakness, atelectasis, insulin resistance, joint contractures, and OH [6–8]. In cardiothoracic and abdominal surgery, postoperative complications remain major clinical problems, despite advances in surgical techniques and perioperative care.

Postoperative OH is characterized by symptoms of dizziness, nausea, vomiting, or syncope during sitting or standing [9]. OH is a well-known clinical complication that can delay early mobilization, although relatively little data are available regarding its mechanism and possible treatment [10]. In addition, the pathophysiology of OH might be related to impaired cardiovascular regulation postoperatively [11], but this relationship is not clearly understood. Previous studies have documented a 12-19% rate of incidence of OH during early postoperative mobilization in patients who were treated for breast cancer, had undergone hip arthroplasty, or had received some type of gynecological treatment [12–15]. Only one study assessed the cardiovascular response and orthostatic intolerance to early mobilization after video-assisted thoracic surgery (VATS). This study demonstrated a 35% incidence of OH [16].

More surgical stress is anticipated in cardiothoracic and abdominal surgery compared to those surgeries. Although, incidence of OH after cardiothoracic and abdominal surgery has not been well-known, we hypothesize that more OH should be identified after cardiothoracic and abdominal surgery. The primary aim of this study was to examine the incidence of OH during early mobilization after major surgery. In addition, we investigated the independent risk factors associated with OH.

## Methods

### Patients

As a prospective observational study, we enrolled patients who underwent cardiac surgery (cardiac group), thoracic surgery (thoracic group), and abdominal surgery (abdominal group) at Nagasaki University Hospital and Seirei Mikatahara General Hospital from October 2008 to January 2012. In each group covered in cardiac group (e.g., coronary heart disease, valve diseases), in thoracic group (e.g., lung cancer, mediastinal neoplasm), in abdominal group (e.g., gastric cancer, liver and pancreatic cancer), respectively. All patients received standard perioperative management and nursing care following the critical path. Patients were included if they were older than 18 years, were undergoing planned surgery, and could provide written informed consent. Patients were established to have a performance status of 0 and to be clinically stable before the surgery. Patients were excluded if they had comorbid conditions that affected exercise performance (e.g., musculoskeletal or neurological impairment); had undergone re-operation; off-pump cardiac surgery; were experiencing atrial fibrillation, vomiting, and diarrhea before mobilization; or had a history of any cerebrovascular disease and postoperative admission to the intensive care unit. The Human Ethics Review Committee of Nagasaki University Hospital (Approval number: 09022760 − 2) and Seirei Mikatahara General Hospital (Approval number: 09-02) approved this study.

### Measurement

A physiotherapist evaluated cardiovascular response and symptoms during an attempt to perform a standing exercise on postoperative day 1 (POD 1). Systolic blood pressure (mmHg; SBP), diastolic blood pressure (mmHg; DBP) and mean arterial pressure (mmHg; MAP), heart rate (beats per minute; HR), oxygen saturation (percentage; SpO_2_), and respiratory rate (frequency per minutes; RR) were monitored throughout mobilization using the BSM-2300 series Life scope-i (Nihon Kohden Corporation, Tokyo, Japan). Their values in a supine position were defined as the baseline levels. We evaluated patients in the supine position, while sitting on the edge of a bed (immediate, 3 and 5 min after), and while standing (immediate and 3 min after). For blood pressure measurements, a brachial cuff was placed around the left arm kept in a fixed position at heart level. HR was measured using an electrocardiogram of the same monitor. SpO_2_ was measured using a probe that attached to the right finger. OH and the cardiovascular response while the patients transitioned from sitting on the edge of the bed to standing were evaluated. Fluid balance was defined volume loss from start of anesthesia to the morning of the day of mobility.

Vasopressors included a postoperative continuous intravenous infusion of catecholamines (i.e., dopamine, dobutamine, epinephrine, and norepinephrine). We calculated the catecholamine index (CAI) [17, 18], as follows, wherein all doses were expressed as μg/kg/min.

CAI = (dopamine dose × 1) + (dobutamine dose × 1) + (epinephrine dose × 100) + (noradrenaline dose × 100).

A selection of perioperative anesthetics and the use of thoracic epidural anesthesia were at the discretion of the attending anesthesiologist. Postoperative analgesics use included continuous intravenous infusion of fentanyl and epidural infusion of ropivacaine hydrochloride. For intravenous infusion of fentanyl, a Terufusion syringe pump TE-331S (TERUMO, Tokyo, Japan) was used. For epidural infusion anesthetics (ropivacaine 0.2%), Infuser SV4 (Baxter Limited, Tokyo, Japan) was used. The basal infusion rate was set at 4 ml/h.

### Assessment of OH

OH was defined as a fall in SBP of at least 20 mmHg or a fall in DBP of at least 10 mmHg within 3 min upon standing and as intolerable dizziness, nausea and vomiting upon mobilization [9, 19]. Mobilization sessions were discontinued when the patients experienced an increase in symptoms until they returned to a sitting position (fainting, excessive pain, dizziness, nausea, sweating, pallor, and postoperative delirium) and when the patients could not measure at baseline vital sign.

### Outcome measures

The primary outcome was to clarify the incidence of OH during early postoperative mobilization after having undergone cardiothoracic and abdominal surgery and the relationship between early mobilization and cardiovascular response changes. The secondary outcome was to identify independent factors associated with OH by performing multivariate logistic regression.

### Statistical analysis

Data were compared using a Wilcoxon’s rank sum test and Fisher’s exact test between cardiothoracic and abdominal groups. Wilcoxon’s rank sum test was performed for continuous variables and Fisher’s exact test was performed for categorical variables. Multivariate logistic regression analysis was then performed to correct for risk factors that showed at least a trend toward significance (*P* < 0.1) in the univariate analysis. Multivariate logistic regression analysis was used to identify the dependent variables of orthostatic hypotension. Data are described as frequencies for categorical variables and as the median and interquartile range (IQR) for quantitative variables. *P*-values <0.05 were considered statistically significant. Statistical analysis was performed using JMP 11.0 software (SAS Institute Japan, Tokyo, Japan).

## Results

**Fig. 1:**
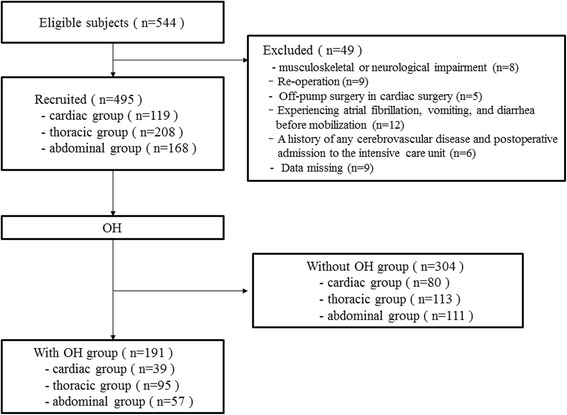
Flow of patients through the study

A total of 544 patients who had undergone cardiothoracic and abdominal surgery were evaluated in a prospective cohort study (Fig. 1). Since data of 49 patients were missing, they were excluded. Table 1 shows the baseline characteristics of the patients. Four hundred ninety-five postoperative patients were categorized into cardiac group (*n* = 119, aged 74 IQR, 66-79 years), thoracic group (*n* = 208, aged 65 IQR, 58-73 years), and abdominal group (*n* = 168, aged 72 IQR, 64-78 years).

**Table 1:** Patient demographics

	Cardiac group (*n* = 119)	Thoracic group (*n* = 208)	Abdominal group (*n* = 168)
Sex, male / female	82 / 37	140 / 68	113 / 55
Age, years	74 (66 to 79)	65 (58 to 73)	72 (64 to 78)
BMI, kg/m^2^	18 (16 to 20)	18 (16 to 20)	18 (16 to 20)
Comorbidities, n (%)			
Cardiovascular	35 (29.4)	50 (24.0)	55 (32.7)
Respiratory	4 (3.4)	38 (18.3)	21 (12.5)
Neurological	5 (4.2)	4 (1.9)	7 (4.2)
Orthopedic	0 (0)	3 (1.4)	20 (11.9)
Preoperative ejection fraction, %	64 (54 to 72)	69 (65 to 73)	71 (65 to 75)
Operative time, min	254 (197 to 323)	252 (186 to 390)	426 (306 to 570)
Operative blood loss, g	700 (400 to 1228)	183 (70 to 446)	600 (275 to 1200)
Blood transfusion, ml	560 (280 to 1120)	0 (0)	0 (0 to 289)
Fluid balance, ml	280 (101 to 569)	413 (148 to 680)	826 (332 to 1336)
Vasopressor, n (%)	65 (54.6)	0 (0)	5 (3.0)
CAI	0.2 (0 to 0.4)	0	0
Analgesia with opioids, n (%)	19 (16.0)	57 (27.4)	141 (83.9)
Epidural anesthesia, n (%)	0 (0)	130 (62.5)	0 (0)
Postoperative hemoglobin, g/dl	10 (10 to 11)	10 (8 to 11)	11 (10 to 12)
Serum creatinine, g/dl	1.0 (0.7 to 1.3)	0.7 (0.5 to 0.8)	0.8 (0.6 to 0.9)
The rate of variability of vital signs			
⊿SBP, %	-2 (6 to −13)	−8 (1 to −18)	−4 (8 to −16)
⊿DBP, %	0 (8 to −11)	−5 (6 to −15)	0 (9 to −8)
⊿MAP, %	−3 (8 to −10)	−7 (3 to −15)	−2 (8 to −10)
⊿HR, %	4 (10 to 0)	9 (15 to 2)	11 (19 to 5)
Initial sitting, day	2 (1 to 3)	1 (1 to 1)	1 (1 to 1)
Initial standing, day	2 (1 to 4)	1 (1 to 1)	1 (1 to 1)

*Notes*: Values were reported as the median (IQR) or number and percentage of subjects

*Abbreviations*: *BMI* Body mass index, *CAI* Catecholamine index, *OH* Orthostatic hypotension, *IQR* Interquartile range, *DBP* Diastolic blood pressure, *HR* Heart rate, *MAP* Mean arterial pressure, *SBP* Systolic blood pressure; ⊿: The range of variation for cardiovascular responses

Coronary heart disease 38 (31.9%), valve diseases 57 (47.9%), aortic and vascular disease 24(20.2%) patients were included in cardiac group. Lung cancer 134 (64.4%), mediastinal neoplasm 12 (5.8%), esophageal cancer 39(18.8%), Other 23 (11.1%) patients were included in Thoracic group. Gastric cancer 33 (19.6%), colorectal cancer 9 (5.4%), liver and biliary system diseases 79 (47.0%), pancreatic cancer 47 (28.0%) patients were included in abdominal group.

**Fig. 2:**
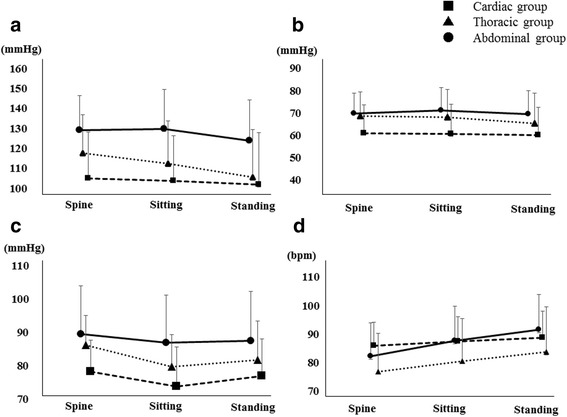
Comparison of the range of variation in cardiovascular response. **a** Systolic blood pressure, {**b**) diastolic blood pressure, (**c**) mean arterial pressure, (**d**) heart rate. OH; orthostatic hypotension

Cardiovascular comorbidities included hypertension in 144 patients (29.1%), arrhythmia in 12 patients (2.4%), and angina pectoris in 5 patients (1.0%). Comorbidities of the respiratory system included chronic obstructive pulmonary disease in 12 patients (2.4%), asthma in 7 patients (1.4%), and interstitial lung disease in 5 patients (1.0%). Neurological system comorbidities included cerebrovascular disease in 5 (1.0%) patients. Orthopedic system comorbidities included vertebral compression fractures and lumbar spinal canal stenosis in 5 (1.0%) patients. Median preoperative ejection fraction in all patients was 70 (65 to 75). Figure 2 shows the cardiovascular response during postoperative mobilization. There were no significant differences in the cardiovascular response between the groups. There were significant differences in type of surgery (*p* = 0.02), analgesia with opioids (*p* = 0.02) and epidural anesthesia (*p* < 0.0001) between those with and without OH (Table 2).

**Table 2:** Demographics of patients who with or without OH

	With OH (*n* = 191)	Without OH (*n* = 304)	*p* Value
Sex, male / female	138 / 53	197 / 107	0.08
Age, years	69 (61 to 77)	70 (61 to 77)	0.81
BMI, kg/m^2^	18 (16 to 20)	18 (16 to 20)	0.57
Comorbidities, n (%)			
Cardiovascular	47 (24.6)	93 (30.6)	0.15
Respiratory	21 (11.0)	42 (13.8)	0.36
Neurological	6 (3.1)	10 (3.3)	0.93
Orthopedic	8 (4.2)	15 (4.9)	0.70
Preoperative ejection fraction, %	67 (63 to 72)	70 (65 to 75)	0.06
Type of surgery, n (%)			0.02
Cardiac surgery	39 (32.8)	80 (67.2)	
Thoracic surgery	95 (45.7)	113 (54.3)	
Abdominal surgery	57 (33.9)	111 (66.1)	
Operative time, min	302 (202 to 438)	305 (2205 to 439)	0.62
Operative blood loss, g	360 (154 to 835)	430 (148 to 805)	0.89
Blood transfusion, ml	0 (0 to 304)	0 (0 to 520)	0.73
Fluid balance, ml	538 (187 to 1093)	442 (125 to 910)	0.18
Vasopressor, n (%)	36 (18.9)	44 (14.8)	0.27
CAI	0 (0 to 0)	0 (0 to 0)	0.13
Analgesia with opioids, n (%)	71 (37.2)	146 (48.0)	0.02
Epidural anesthesia, n (%)	74 (38.7)	56 (18.4)	< .0001
Postoperative hemoglobin, g/dl	11 (10 to 12)	11 (10 to 12)	0.74
Serum creatinine, g/dl	0.8 (0.6 to 1.0)	0.8 (0.6 to 1.0)	0.42
Initial sitting, day	1 (1 to 2)	1 (1 to 2)	0.94
Initial standing, day	1 (1 to 2)	1 (1 to 2)	0.28

*Notes*: Values were reported as the median (IQR) or number and percentage of subjects

*Abbreviations*: *BMI* Body mass index, *CAI* Catecholamine index, *OH* Orthostatic hypotension, *IQR* Interquartile range, *DBP* Diastolic blood pressure, *HR* Heart rate, *MAP* Mean arterial pressure, *SBP* Systolic blood pressure

**Fig. 3:**
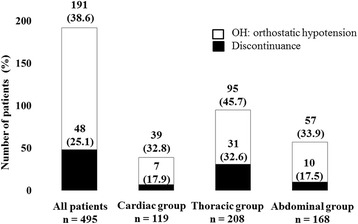
Incidence rate of OH and rate of discontinuation during postoperative mobilization

One hundred and ninety-one (38.6%) of 495 patients exhibited OH. The incidence of OH was 39 (32.8%) of 119 in cardiac patients, 95 (45.7%) of 208 in thoracic patients, and 57 (33.9%) of 168 in abdominal patients. Forty-eight (25.1%) of 191 patients withdrew during postoperative mobilization. Of these 48 patients, seven (17.9%) of 39 patients had undergone cardiac surgery, 31 (32.6%) of 95 patients had undergone thoracic surgery, and 10 (17.5%) of 57 patients had undergone abdominal surgery (Fig. 3). Early mobilization was discontinued in 48 patients due to dizziness in 16 patients (33.3%), nausea in 15 patients (31.3%), wound pain in 9 patients (18.6%), and fatigue in 8 patients (16.7%). There was no significant correlation between discontinuation and the rate of variability in SBP (*p* = 0.74), DBP (*p* = 0.28), HR (*p* = 0.50) and MAP (*p* = 0.52).

There were significant differences in blood transfusion (*p* = 0.01), vasopressor (*p* = 0.02) and CAI (*p* = 0.01) between those with and without OH in the cardiac group. There were significant differences in fluid balance (*p* = 0.01), analgesia with opioids (*p* < 0.05) and epidural anesthesia (*p* < 0.001) between those with and without OH in the thoracic group. There were significant differences in operative time (*p* = 0.02), operative blood loss (*p* = 0.01), blood transfusion (*p* < 0.05), vasopressor (*p* < 0.05), and initial standing (*p* = 0.02) between those with and without OH in the abdominal group (Table 3).

**Table 3:** Demographics of patients in type of surgery who with or without OH

	Cardiac group			Thoracic group			Abdominal group		
	With OH	Without OH	*p*-value	With OH	Without OH	*p*-value	With OH	Without OH	*p*-value
	(*n* = 39)	(*n* = 80)		(*n* = 95)	(*n* = 113)		(*n* = 57)	(*n* = 111)	
Sex, male / female	8 / 31	51 / 29	0.08	68 / 27	72 / 41	0.23	39 / 18	74 / 39	0.82
Age, years	72 (61 to 78)	75 (69 to 79)	0.06	67 (60 to 73)	64 (57 to 72)	0.08	72 (67 to 78)	71 (63 to 78)	0.45
BMI, kg/m^2^	18 (17 to 19)	18 (16 to 20)	0.72	18 (16 to 20)	18 (16 to 20)	0.97	18 (15 to 19)	18 (16 to 20)	0.50
Comorbidities, n (%)									
Cardiovascular	12 (31.0)	27 (33.8)	0.82	17 (17.9)	33 (29.2)	0.07	18 (31.6)	37 (33.3)	0.86
Respiratory	0	4 (5.0)	0.07	14 (14.7)	24 (21.2)	0.23	7 (12.3)	14 (12.6)	0.95
Neurological	1 (2.6)	4 (5.0)	0.52	2 (2.1)	2 (1.8)	0.86	3 (5.3)	4 (3.6)	0.69
Orthopedic	0	0		2 (2.1)	1 (0.9)	0.67	7 (12.3)	13 (11.7)	0.91
Preoperative ejection fraction, %	63 (57 to 70)	67 (53 to 73)	0.53	67 (62 to 73)	71 (63 to 75)	0.14	65 (60 to 69)	68 (62 to 74)	0.07
Operative time, min	227 (190 to 316)	263 (199 to 331)	0.14	252 (192 to 318)	252 (180 to 486)	0.52	492 (366 to 570)	390 (264 to 564)	0.02
Operative blood loss, g	575 (285 to 848)	750 (470 to 1420)	0.06	180 (85 to 460)	189 (52 to 405)	0.38	870 (330 to 1810)	515 (181 to 915)	0.01
Blood transfusion, ml	350 (188 to 708)	730 (280 to 1150)	0.01	0 (0 to 0)	0 (0 to 0)	0.40	0 (0 to 560)	0 (0 to 0)	< 0.05
Fluid balance, ml	591 (246 to 1267)	607 (242 to 1104)	0.77	592 (299 to 1218)	441 (109 to 1007)	0.01	347 (104 to 650)	322 (111 to 631)	0.70
Vasopressor, n (%)	27 (69.2)	38 (47.5)	0.02	5 (5.3)	5 (4.4)	0.78	4 (7.0)	1 (0.9)	< 0.05
CAI	0.3 (0 to 0.5)	0 (0 to 0.3)	0.01	0 (0 to 0)	0 (0 to 0)	0.77	0 (0 to 0)	0 (0 to 0)	–
Analgesia with opioids, *n* (%)	6 (15.4)	13 (16.3)	0.90	16 (16.8)	41 (36.3)	< 0.05	49 (86.0)	94 (84.7)	0.83
Epidural anesthesia, n (%)	0	0		0 (0 to 0)	56 (49.6)	< 0.001	0	0	
Postoperative hemoglobin, g/dl	10.1 (9.5 to 11.1)	10.3 (9.6 to 11.6)	0.41	11.7 (10.7 to 12.8)	11.9 (10.8 to 13.1)	0.21	10.7 (10.0 to 11.4)	10.8 (8.8 to 14.4)	0.94
Serum creatinine, g/dl	0.9 (0.7 to 1.4)	1.0 (0.7 to 1.3)	0.77	0.7 (0.5 to 0.8)	0.7 (0.6 to 0.8)	0.93	0.8 (0.6 to 1.0)	0.8 (0.6 to 0.9)	0.83
Initial sitting, day	2 (1 - 4)	2 (1 - 3)	0.87	1 (1 - 1)	1 (1 - 1)	0.67	1 (1 - 1)	1 (1 - 2)	0.06
Initial standing, day	2 (2 - 4)	2 (1 - 3)	0.37	1 (1 - 1)	1 (1 - 1)	0.35	1 (1 - 1)	1 (1 - 2)	0.02

*Notes*: Values were reported as the median (IQR) or number and percentage of subjects

*Abbreviations*: *BMI* Body mass index, *CAI* Catecholamine index, *OH* Orthostatic hypotension, *IQR* Interquartile range

According to the postoperative medication received, antihypertensive agents were prescribed to 29 (24.4%) patients, antiarrhythmic agents to 24 (20.2%) patients in cardiac group. These medications were no significant differences in the cardiovascular response between the groups. Nineteen (16.0%) of 119 patients in the cardiac group received postoperative continuous intravenous infusion of fentanyl, with the median rate of infusion being 25 μg/h (IQR, 25-50 μg/h). Of the 208 thoracic surgery patients, 130 (62.5%) patients received a continuous postoperative epidural infusion of ropivacaine, with the median rate of infusion being 8 μg/h, and 57 (27.4%) received fentanyl, with the median rate of infusion being 50 μg/h (IQR, 50-50 μg/h). Of the 168 abdominal patients, 141 (83.9%) received fentanyl with the median rate of infusion being 50 μg/h (IQR, 28-50 μg/h).

In a univariate analysis of the following parameters, the type of surgery, sex, age, body mass index, preoperative ejection fraction, fluid balance, operative time, blood loss, the value of hemoglobin and serum creatinine measured on POD 1, CAI, the use of vasopressors, analgesia with opioids, and epidural anesthesia showed a significant relationship with OH.

Multivariate logistic regression analysis indicated an independent association between the incidence of OH and male sex (Odds ratio (OR); 1.538, 95% CI, 1.027 to 2.326; *p* = 0.03) or receiving epidural anesthesia (OR; 2.906, 95% CI, 1.924 to 4.419; *p* < 0.001; Table 4).

**Table 4:** Results for determining factors of OH

Variable	Unadjusted			Adjusted		
	OR	(95% CI)	*p* Value	OR	(95% CI)	*p* Value
Age	0.10	(0.966 to 1.026)	0.83			
BMI	1.08	(0.974 to 1.207)	0.15			
Sex (Male = 1, Female = 0)	2.346	(1.170 to 4.705)	0.01	1.538	(1.027 to 2.326)	0.03
Type of surgery						
Thoracic surgery	1.0 [Reference]					
Cardiac surgery	1.491	(0.453 to 4.909)	0.59			
Abdominal surgery	1.933	(0.810 to 4.615)	0.14			
Preoperative ejection fraction	1.019	(0.992 to 1.050)	0.21			
Operative time	0.984	(0.875 to 1.109)	0.79			
Operative blood loss	1.000	(1.000 to 1.000)	0.97			
Fluid balance, ml	0.100	(1.000 to 1.000)	0.85			
Vasopressor (with = 1, without = 0)	0.425	(0.151 to 1.197)	0.53			
Analgesia with opioids (with = 1, without = 0)	1.433	(0.615 to 3.338)	0.40			
Epidural anesthesia (with = 1, without = 0)	4.329	(1.064 to 17.617)	0.04	2.906	(1.924 to 4.419)	< 0.001
Postoperative hemoglobin	0.917	(0.735 to 1.140)	0.41			

*Abbreviations*: 95% CI: 95% confidence interval, *BMI* Body mass index, *CAI* Catecholamine index, *OR* Odds ratio, *OH* Orthostatic hypotension

## Discussion

The main findings of the present study in patients who had undergone cardiothoracic and abdominal surgery, we found that (1) 38.6% experienced OH during early mobilization after surgery and (2) men and patients who received an epidural anesthesia were predisposed to experience OH during early mobilization.

The results suggest that the range of variation in the cardiovascular responses during postoperative mobilization is limited, and we did not recognize an association between the cardiovascular response and symptoms. In general, postoperative patients are particularly vulnerable upon mobilization aggravate the postural reduction in central blood volume in the upright position [20]. However, a previous study also indicated that there was no relationship between postoperative symptoms and cardiovascular response during early mobilization [21]. Our results were consistent with those of the previous report. Therefore, it is important to assess the subjective symptoms of patients in addition to measuring cardiovascular response during mobilization.

The incidence of OH and the use of postoperative opioids were higher than in previous studies [12–16], and a quarter of patients discontinued from the study. Approximately 60% of cases that discontinued early mobilization were due to dizziness and nausea. In general, the use of postoperative opioids is associated with an increased risk of nausea and vomiting, thus resulting in an increase in the incidence and severity of OH [15, 22]. Although it is reasonable to assume that both epidural analgesia and analgesia with opioids are the causes of OH, we found that only epidural anesthesia was associated with an increased risk of OH. The use of epidural anesthetics theoretically induces OH through sympathectomy-induced vasodilation [16]. Gramigni E et al. [23] reported cardiovascular response during postoperative mobilization that involved the use of thoracic epidural analgesia with a mixture of bupivacaine and fentanyl. They concluded that epidural analgesia was associated with arterial hypotension during the postoperative period. Although we agree with their opinion, our results identified that epidural analgesia without opioids was an independent predisposing factor for OH. However, it is difficult to be certain of the relationship between epidural analgesia and OH based upon the results of this study only. Overall, when employing early mobilization, clinicians and physiotherapists should be careful when postoperative patients use analgesics to treat OH. As discussed above, many studies offer different perspectives; however, it is approximately certain that analgesics influence the incidence of OH. Since most patients use analgesics after surgery, clinicians should take precautions when mobilizing these patients. Furthermore, physiotherapists should advise patients to be in Fowler’s position and wear elastic stockings during the day to reduce the risk of OH [24]. Early mobilization using various devices is important for prevention of orthostatic intolerance, deep vein thrombosis and pulmonary complications.

In the cardiac group, rigorous postoperative management was required to prevent heart failure and postoperative bleeding such as open aortic surgery and heart valve surgery. In this study, 55% of vasopressor use occurred in the cardiac group. When we evaluated the rate of OH and discontinuation in the cardiac group, the use of vasopressor might not have influenced the relationship between orthostatic cardiovascular responses, although vasopressor use and the CAI were significantly different between the groups with and without OH. It is reasonable for us to anticipate that the incidence of OH would be increasing without vasopressor use; however, in our results, OH was more prevalent in patients using vasopressors. Therefore, it was difficult to assess the influence of vasopressor use in the cardiac surgery group. Also, antihypertensive and antiarrhythmic agents were used in cardiac group. Although, these medicines were no significant differences in the cardiovascular response between the groups, we should continue to evaluate the relationship between these medicines use and OH development.

OH was observed more frequently in the thoracic group. Epidural anesthesia was shown to be an independent predisposing factor for OH development in multivariate analysis, and it was used only in this group. Mizota et al. [16] found that postoperative opioid use were independent risk factors for OH after VATS. In addition, they demonstrated that continuous postoperative epidural administration of ropivacaine at 0.2% at a rate of 2-6 ml/h did not induce clinically significant vasodilation. In our study, we applied epidural anesthesia using a continuous postoperative epidural administration of the same dose of ropivacaine. In doing so, we found that its use was an independent predisposing factor for OH. Thus, we observed a different result from that of the previous study. Therefore, in the future, we will attempt to clarify the influence of epidural anesthesia on the development of OH.

In the abdominal group, operative factors might not be associated with OH between patients with and without OH. Haines KJ et al. [25] concluded that 52% of patients undergone high-risk abdominal surgery had a barrier to mobilization, with the most common barrier being hypotension. In our study, we did not detect a relationship between blood pressure and OH. It was difficult to anticipate OH using only the cardiovascular response in patients who had undergone abdominal surgery. Future studies should include physiological parameters to predict the incidence of OH in patients who had undergone abdominal surgery.

The male was independently associated with the incidence of OH. Convertino [26] demonstrated that fluid volume shifting during postural changes had been lower in females than in males. Females are at an increased risk for OH as a result of common variables related to body size and hormones [27]. However, our results did not show similar results, which may possibly be due to two reasons. First, our subjects were postoperative patients. Surgical trauma imposes increased demands on organs with increased sympathetic tone and a subsequent endocrine metabolic response. In addition, blood loss and fluid volume shifts that might occur might influence the cardiovascular response to mobilization [20]. Second, in our subjects, more males were enrolled than females. These differences may also be related to muscle volume and body size. Therefore, our results suggest that male postoperative patients might experience orthostatic hypotension due to increased muscle sympathetic and peripheral blood vessel outflow due to muscle contraction. Currently, sex differences in OH have been observed, but the mechanisms are still unknown [27].

This study had several limitations. First, we investigated different types of surgeries because we wanted to capture the characteristics of each surgery. The mechanism of fluid shifts of each groups are probably heterogeneous due to complicated and diverse group of patients. However, the reason for targeting patients at postoperative course of thoracic and abdominal surgery is justified because these surgeries have an influence on autonomic nervous system and OH of postoperative complications are most likely to occur. Therefore, it was considered important to assess the cardiovascular response and orthostatic intolerance during mobilization after these postoperative surgeries. Second, we evaluated the phenomenon of hypotension under a clinical setting. Anatomical physiological details were unknown. Further studies are necessary to clarify these limitations.

## Conclusion

The present study found a high incidence of OH during early mobilization in patients who had undergone cardiothoracic and abdominal surgery. Two independent predisposing factors for OH (male sex, and epidural anesthesia in thoracic surgery) were identified. In conclusion, it should be recognized that OH occurs frequently during postoperative early mobilization.

## Abbreviations

CAI: Catecholamine index
DBP: Diastolic blood pressure
ERAS: Enhanced recovery after surgery
HR: Heart rate
IQR: Interquartile range
MAP: Mean arterial pressure
OH: Orthostatic hypotension
POD: Postoperative day
PPCs: Postoperative pulmonary complications
RR: Respiratory rate
SBP: Systolic blood pressure
SpO2: Oxygen saturation
VATS: Video-assisted thoracic surgery
